# Analysis of factors affecting acute pain after shoulder arthroscopy: a retrospective clinical study

**DOI:** 10.1051/sicotj/2026085

**Published:** 2026-10-12

**Authors:** Ziyu Wang, Bin Fang, Lan Wei, Yuanyuan Qi, Chao Gong, Lina Huang

**Affiliations:** Department of Anesthesiology, Shanghai General Hospital, Shanghai Jiao Tong University School of Medicine No. 650, New Songjiang Road Shanghai 201620 China.

**Keywords:** Postoperative pain, Shoulder arthroscopy, Apolipoprotein A-I, Nerve block

## Abstract

*Background*: Arthroscopic shoulder surgery is a minimally invasive treatment; however, patients still experience significant postoperative pain. This study aimed to identify factors related to acute pain after shoulder arthroscopy and guide pain management. *Methods*: This study included 272 patients who underwent shoulder arthroscopic surgery at our hospital between January 2020 and August 2021. Blood samples for apolipoprotein measurement were collected on the day before surgery after an overnight fast. Pain was assessed using the VAS at 24 h after surgery as the primary endpoint. Patients were divided into two groups according to pain levels 24 h after surgery: mild pain (VAS ≤3) and moderate-to-severe pain (VAS > 3). Logistic regression was used to analyze the relationships between postoperative pain and age, sex, BMI, injury type, method of operation, nerve block, and apolipoprotein levels. *Results*: The majority of patients (71.32%, 194/272) had moderate- to severe postoperative pain. Analysis revealed significant differences between the two groups in surgical method (*P* = 0.009), injury type (*P* = 0.042), nerve block administration (*P* = 0.039), apolipoprotein A-I levels (*P* = 0.006), and apolipoprotein C (*P* = 0.046). Logistic regression analysis showed that postoperative pain was correlated with nerve block administration and apolipoprotein A-I levels. *Conclusion*: The absence of nerve block and elevated apolipoprotein A-I levels were associated with more severe postoperative pain after shoulder arthroscopy. Therefore, the findings suggest that brachial plexus block may reduce postoperative pain. Patients with elevated ApoA-I may benefit from closer monitoring.

## Introduction

Arthroscopic shoulder surgery is widely performed in clinical practice. However, shoulder surgery for rotator cuff syndrome is associated with significant postoperative pain [1], which may impede recovery and functional rehabilitation [2, 3]. Patients with preoperative pain who took non-steroidal anti-inflammatory drugs and did not receive nerve blocks experienced more intense pain within 24 h after surgery [4]. Patients undergoing rotator cuff repair and stabilization had higher pain scores 24 h–1 month after surgery. In addition, the degree and frequency of preoperative pain influence postoperative pain, particularly in young and female patients with work-related injuries [5]. Patients undergoing shoulder arthroscopic rotator cuff repair experience varying degrees of pain for days or even weeks after surgery, posing challenges for both patients and surgeons [6]. Patients with small rotator cuff tears have less postoperative pain than those with large tears. Previous studies [7, 8] have shown that intraoperative findings of superior labrum, anterior and posterior injuries involving the biceps tendon are associated with greater postoperative opioid requirements.

Serum lipid markers have been linked to chronic pain, with certain metabolites potentially involved in inflammatory pathways common to pain and obesity [9]. Obesity and hyperlipidemia are established risk factors for knee osteoarthritis and rotator cuff injury, and they may also impede postoperative recovery [10–13]. Whether specific lipoprotein components directly affect surgical pain outcomes, however, remains unclear.

Apolipoprotein A-I (ApoA-I), the major HDL protein, is conventionally viewed as a hyperlipidemia marker [14]. Beyond cholesterol transport, ApoA-I exhibits anti-inflammatory properties, including endotoxin neutralization and macrophage modulation that could theoretically attenuate the local inflammatory response to surgical trauma. Given that shoulder arthroscopy induces substantial soft tissue inflammation driving early postoperative pain, ApoA-I represents a biologically plausible candidate for investigation. However, clinical evidence on this association is lacking. In contrast, apolipoprotein C has been associated with central pain conditions and fibromyalgia [15], but its relevance to acute peripheral postsurgical pain is uncertain. Few studies have examined either apolipoprotein in relation to postoperative pain [16].

We therefore adopted an exploratory approach, focusing on ApoA-I as a primary variable to generate preliminary evidence. At present, the clinical management of postoperative pain remains imperfect; therefore, identifying factors affecting pain after shoulder arthroscopy is of considerable importance. This study aimed to explore factors associated with postoperative pain in patients undergoing shoulder arthroscopy, with the goal of informing intraoperative and postoperative pain management strategies.

## Methods

### Patient selection

This was a retrospective cohort study of patients who underwent shoulder arthroscopic surgery under general anesthesia at our hospital between January 2020 and August 2021. All surgeries were performed by a single experienced surgeon. This study was approved by our hospital's institutional review board and registered at the China Clinical Trial Center. Written informed consent was obtained from all patients and/or their families. Patient confidentiality and data protection were ensured in accordance with institutional policies.

Inclusion criteria:

1. Age ≥ 18 years;
2. ASA I–II;
3. Elective unilateral shoulder arthroscopic surgery, including rotator cuff repair or debridement;

Exclusion criteria:

1. Revision or bilateral surgery;
2. Pre-existing neurological disorders or inflammatory arthritis as documented in medical records;
3. Contraindications to regional anesthesia;
4. Conversion to open surgery;
5. Failed or incomplete nerve block;
6. Incomplete data on key variables (weight, VAS score, nerve block status, or apolipoprotein A-I level).

Patients were included consecutively during the study period if they met the above criteria. A total of 293 patients were initially screened, of whom 21 were excluded due to incomplete key data (*n* = 21). The remaining 272 patients were included in the final analysis. The patient selection process is illustrated in Figure 1.

**Figure 1:**
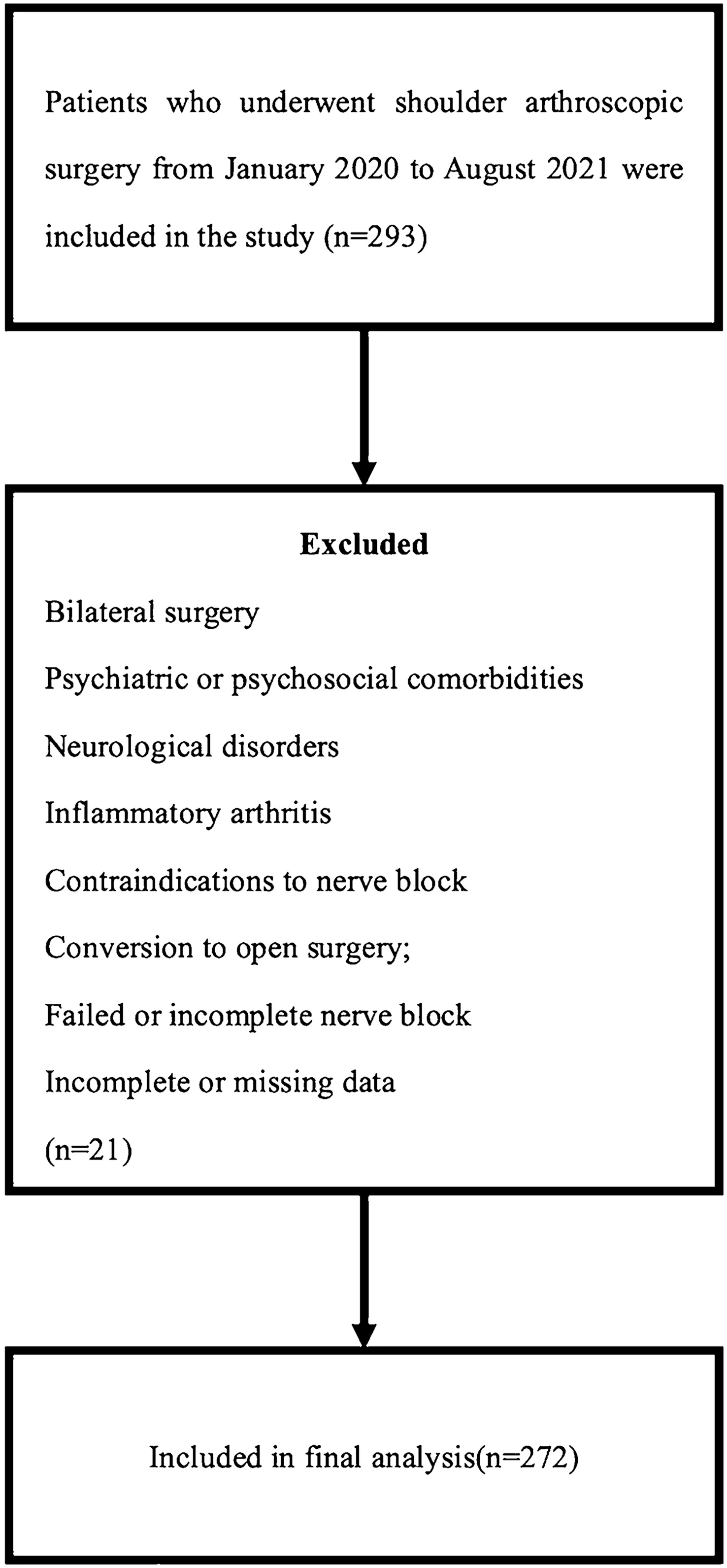
Patient selection flow diagram. A total of 293 patients were initially screened; 21 patients were excluded due to incomplete key data. The final analysis included 272 patients who underwent shoulder arthroscopic surgery.

### Methods

Patients underwent routine fasting and abstained from liquids before surgery. After the patient entered the operating room, routine non-invasive blood pressure, electrocardiogram, and oxygen saturation monitoring were performed. All patients underwent general anesthesia combined with a regional block. After routine monitoring and preoxygenation, an ultrasound-guided interscalene brachial plexus block was performed by an experienced attending anesthesiologist prior to the induction of general anesthesia. A linear high-frequency ultrasound probe was used to identify the brachial plexus trunks between the anterior and middle scalene muscles at the level of the cricoid cartilage. A total of 15 mL of 0.25% ropivacaine was injected in fractionated doses under real-time visualization to ensure adequate spread around the superior and middle trunks. Block success was confirmed by the presence of sensory blockade in the C5–C6 dermatomes (deltoid and lateral arm) using a cold test within 15 minutes after injection. General anesthesia was then induced with propofol (2 mg/kg), sufentanil (0.3 μg/kg), and rocuronium (0.6 mg/kg), and maintained with desflurane (1.0–1.3 MAC) in an oxygen/air mixture. Additional rocuronium and sufentanil were administered as needed to maintain stable surgical conditions. At the end of the procedure, residual neuromuscular blockade was reversed with neostigmine and atropine, and the trachea was extubated after the patient regained consciousness. Then the patient was moved to the postanesthesia care unit. General data included gender, age, height, weight, BMI, preoperative underlying diseases, and laboratory test results (apolipoproteins A-I and C). Blood samples for laboratory tests, including apolipoprotein A-I and apolipoprotein C, were collected on the day before surgery after an overnight fast, prior to any fluid or medication administration. Apolipoprotein A-I and apolipoprotein C levels were measured using immunoturbidimetric assays with commercially available reagents. The assays were calibrated using manufacturer-provided calibrators, and internal quality control samples were run with each batch**.** Intraoperative data included operation duration, method of operation, shoulder joint injury type, and nerve block status. Postoperative pain at rest was assessed using the Visual Analog Scale (VAS) at 24 h after surgery as the primary endpoint. In addition, as part of our extended follow-up protocol, VAS scores were also recorded at regular intervals during the remainder of the hospital stay until discharge. However,because the 24 h time point provided the most complete and standardized data across all patients and given that discharge timing varied considerably, the primary analysis focused on VAS score at 24 h after surgery. VAS scores were recorded by trained nursing staff who were not involved in the study and were not specifically blinded to the nerve block status. Pain scores were assessed before the administration of rescue analgesia when possible. According to the VAS score, patient data were divided into two groups: mild pain (VAS ≤ 3) and moderate-to-severe pain (VAS > 3). Through data collection and statistical analyses, the factors affecting acute pain after shoulder arthroscopy were analyzed. SPSS (version 26.0) was used for the statistical analysis of relevant risk factors. Continuous data were presented as mean ± SD and compared using the independent samples *t*-test; categorical data were presented as *n* (%) and compared using the Chi-square test or Fisher's exact test, as appropriate. Variables with *P* < 0.1 in univariate analysis were entered into a multivariate logistic regression model to identify independent factors associated with moderate-to-severe postoperative pain. *P* < 0.05 values were considered to be statistically significant. The research process is illustrated in Figure 1.

## Results

### Patient characteristics

Data were extracted from the hospital medical records and entered into SPSS (version 26.0). Missing data were handled by complete-case analysis, as only 21 of 293 screened patients (7.2%) had incomplete key variables and were excluded. No imputation was performed. A total of 272 patients were included in the final analysis. The baseline and perioperative characteristics of the mild pain and moderate-to-severe pain groups are presented in Table 1. Continuous variables were compared using the independent samples *t*-test and expressed as mean ± SD. Categorical variables were compared using the Chi-square test (or Fisher's exact test when expected cell counts were < 5) and expressed as *n* (%). Significant differences between the two groups were observed in surgical method (*P* = 0.009), injury type (*P* = 0.042), nerve block administration (*P* = 0.039) apolipoprotein A-I levels (*P* = 0.006), and apolipoprotein C levels (*P* = 0.046).

**Table 1:** Baseline and perioperative characteristics of patients according to pain group (*n* = 272).

Variables	Mild pain group (*n* = 78)	Moderate to severe pain group (*n* = 194)	*P* value
Sex, *n* (%)			0.280
Male	19 (24.4)	60 (30.9)	
Female	59 (75.6)	134 (69.1)	
Height (m)	1.6 ± 0.08	1.6 ± 0.07	0.417
Weight (kg)	63.9 ± 10.6	64.1 ± 10.5	0.867
BMI (kg/m^2^)	24.9 ± 3.8	24.7 ± 3.2	0.628
Age (yr)	59.3 ± 10.1	57.1 ± 9.4	0.047*
Diabetes, *n* (%)	9 (11.2)	16 (8.2)	0.396
High blood pressure, *n* (%)	16 (20.5)	34 (17.5)	0.565
Coronary heart disease, *n* (%)	1 (1.3)	4 (2.1)	0.665
Course of disease			0.654
0–90 days	23 (29.5)	52 (26.8)	
>90 days	55 (70.5)	142 (73.2)	
Method of surgery			0.009**
Debridement	5 (6.4)	37 (19.1)	
Repair	73 (93.6)	157 (80.9)	
Injury types			0.042^*^
Type I or II	32 (41.0)	106 (54.6)	
Type III or IV	46 (59.0)	88 (45.4)	
Nerve block, *n* (%)			0.039
Yes	40 (51.3)	73 (37.6)	
No	38 (48.7)	121 (62.3)	
Opioid usage (ug)	23.7 ± 6.9	24.4 ± 6.2	0.396
Duration of operation (min)	92.6 ± 27.0	93.9 ± 27.6	0.726
Remifentanil			0.338
Yes	25 (32.1)	51 (26.3)	
No	53 (67.9)	143 (73.7)	
Other analgesics			0.903
Yes	12 (15.4)	31 (16.0)	
No	66 (84.6)	163 (84.0)	
Apolipoprotein A-I (mg/dL)	26.1 ± 4.1	28.5 ± 7.4	0.006**
Apolipoprotein C (mg/dL)	5.3 ± 3.2	4.4 ± 3.3	0.046*

**Table 2:** Multivariable logistic regression analysis of factors associated with moderate-to-severe pain (VAS > 3) at 24 h after shoulder arthroscopy (*n* = 272).

Variable	*P* value	OR	95% CI
Age	0.798	0.996	0.963–1.029
Sex	0.845	0.992	0.911–1.079
BMI	0.556	0.821	0.426–1.583
Surgical method	0.053	0.355	0.124–1.013
Injury type	0.016	0.473	0.26–0.859
Nerve block	0.016	2.07	1.15–3.73
ApoA-I	0.001	1.103	1.040–1.170
ApoC	0.166	0.936	0.852–1.028

Model fit: Omnibus test: χ^2^ = 32.047, df = 10, *P* < 0.001; Hosmer-Lemeshow test: χ^2^ = 9.167, df = 8, *P* = 0.328; Nagelkerke *R*^2^ = 0.159.

### Factors related to postoperative pain

Multivariable logistic regression analysis was performed to identify independent factors associated with moderate-to-severe pain at 24 h after surgery. Variables with *P* < 0.1 in univariate analysis, along with clinically important variables (age, sex, BMI, surgical method, injury type, nerve block status, apolipoprotein A-I, and apolipoprotein C), were entered into the model using the Enter method.

The results are presented in Table 2 and Figure 2. The model was statistically significant (Omnibus χ^2^ = 32.047, df = 10, *P* < 0.001) and demonstrated good fit (Hosmer-Lemeshow χ^2^ = 9.167, df = 8, *P* = 0.328). The model explained 15.9% of the variance in postoperative pain (Nagelkerke *R*^2^ = 0.159).

**Figure 2:**
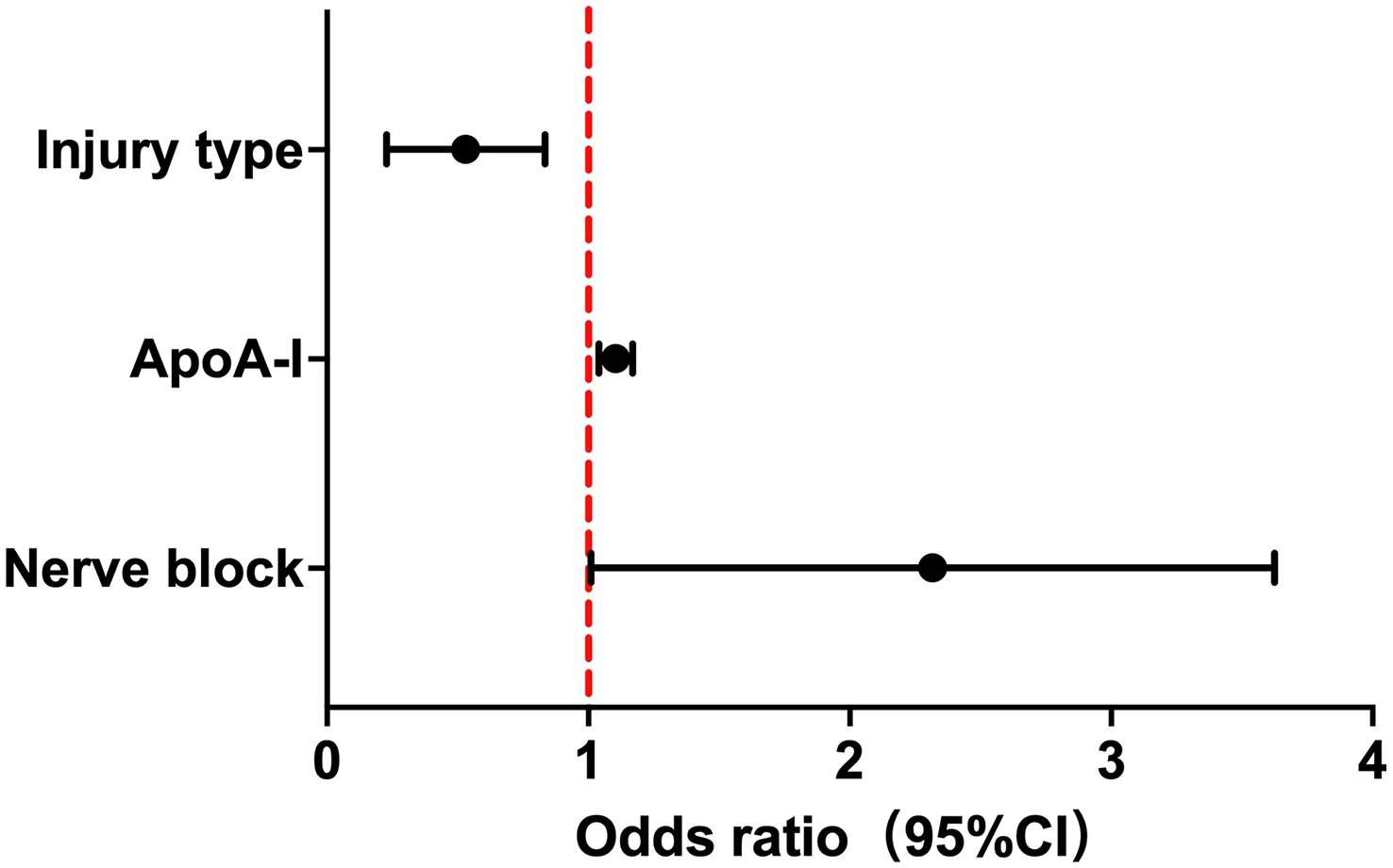
Forest plot of multivariable logistic regression for factors associated with moderate-to-severe pain (VAS > 3) at 24 h post-surgery (*n* = 272). Adjusted ORs and 95% CIs are shown on a log scale. Dashed vertical line indicates OR = 1.0 (no effect). Abbreviations: CI, confidence interval; OR, odds ratio; VAS, visual analog scale.

After adjusting for all covariates, the absence of a nerve block (adjusted OR = 2.07, 95% CI: 1.15–3.73, *P* = 0.016) and higher apolipoprotein A-I levels (adjusted OR = 1.103 per mg/dL increase, 95% CI: 1.040–1.170, *P* = 0.001) remained independently associated with a greater likelihood of moderate-to-severe postoperative pain. Injury type was also identified as a significant factor (adjusted OR = 0.473, 95% CI: 0.260–0.859, *P* = 0.016), indicating that patients with Type III or IV injuries had a lower risk of moderate-to-severe pain compared to those with Type I or II injuries. Other variables, including age, sex, BMI, surgical method, and apolipoprotein C, were not statistically significant in the multivariable model (all *P* > 0.05).

## Discussion

Shoulder arthroscopy is widely performed as a minimally invasive treatment. Owing to its reduced trauma and quick recovery, it is favored by both surgeons and patients. However, as shoulder arthroscopy has become more widely used, acute postoperative pain has gained increasing attention. This retrospective study aimed to identify factors associated with acute pain after shoulder arthroscopy to inform perioperative pain management.

In this study, 28.68% (78/272) of patients experienced mild pain (VAS ≤ 3 points), and 71.32% (194/272) were in the moderate to severe pain group (VAS > 3 points). This was consistent with the clinically observed phenomenon, that is, most patients experience moderate-to-severe pain after shoulder arthroscopy. Therefore, studying the factors influencing the occurrence of acute pain in patients undergoing shoulder arthroscopy is of great clinical significance.

Compared with general anesthesia alone, a brachial plexus block provides better postoperative pain management and reduces the use of opioids after upper extremity surgery [17, 18]. Ultrasound guidance improves block success and reduces nerve injury risk. Our results showed that patients who did not receive an interscalene brachial plexus block experienced more severe postoperative pain, consistent with previous findings [4]. However, given the retrospective, non-randomized design, the decision to administer a nerve block likely depended on clinical factors such as comorbidities, anticoagulation status, or anticipated surgical complexity, creating potential confounding by indication. Therefore, our data suggest that brachial plexus block use is associated with improved early pain control, but this association should not be interpreted as causal.

There were no significant differences in opioid use between groups in this study. Biceps tenotomy and subacromial decompression were risk factors for increased postoperative pain (NRS score > 7) [8]. However, it has also been confirmed that there are more neural networks in the joint capsule, with the majority being free nerve endings. Subacromial decompression was performed to remove more nerve endings; therefore, patients experienced less postoperative pain. Although there are also free nerve endings in tendons, there has been little research on muscle and tendon pain [19]. The types of injuries in this study were defined based on the classification of the Southern California Orthopedic Research Institute [20]. The results of this study showed that more patients in the moderate-to-severe group underwent single tendon or simple cleaning, and multivariate regression analysis showed no significant differences between the two groups in surgery type. Clinically, the choice of surgical procedure is largely dictated by the underlying injury type, making it difficult to disentangle their individual effects on postoperative pain in observational studies. Therefore, more studies are needed to confirm the effects of the injury type and surgical method on postoperative pain.

Apolipoprotein A-I (ApoA-I) is the major protein component of high-density lipoprotein (HDL) and plays a central role in reverse cholesterol transport [14]. Beyond its lipid-related functions, emerging evidence suggests that ApoA-I may participate in pain modulation. Specifically, the ApoA-I binding protein (AIBP) has been shown to reduce neuroinflammation by disrupting TLR4-enriched lipid rafts in spinal microglia, thereby reversing established neuropathic pain states in animal models [21].

Our study found that higher preoperative serum ApoA-I levels were independently associated with a greater likelihood of moderate-to-severe pain at 24 h after shoulder arthroscopy. To our knowledge, this is one of the few studies to explore the relationship between ApoA-I and acute postoperative pain. Importantly, we collected blood samples for apolipoprotein measurement the day before surgery after an overnight fast, ensuring the measured levels reflect the patient’s baseline metabolic status and are not influenced by perioperative hemodilution or the acute-phase inflammatory response to surgical trauma. This preoperative sampling timing is a strength of our study, as it rules out reverse causation (postoperative pain influencing the biomarker).

Previous studies have shown that lipid profiles correlate with pain severity in chronic pain conditions, including knee osteoarthritis [22, 23] and spinal cord injury-related pain [24]. However, most of these studies focused on cholesterol and lipoproteins, with limited attention to apolipoproteins. A study on sickle cell disease reported that ApoA-I levels decreased significantly during acute painful episodes, suggesting that apolipoproteins may fluctuate in response to pain or inflammation [25]. In contrast, our study measured ApoA-I preoperatively and found that higher baseline levels were associated with more severe postoperative pain. This discrepancy may reflect differences between chronic pain states and acute postoperative pain, or between the underlying disease processes.

The positive association between preoperative ApoA-I levels and more severe postoperative pain observed in our study appears to contrast with previous findings linking higher HDL–C to reduced chronic pain risk. Using NHANES data from 4,688 American adults，the study found that each 20 mg/dL increase in HDL–C was associated with a 26% reduction in chronic pain risk, and the highest HDL–C quartile had a 24% lower risk compared with the lowest quartile [26]. ApoA-I is the major structural protein of HDL, and this discrepancy may reflect fundamentally different roles of the ApoA-I/HDL axis in acute versus chronic pain states. In chronic conditions such as knee osteoarthritis, dyslipidemia is associated with a higher prevalence of rotator cuff disease (pooled OR = 2.17, 95% CI: 1.46–3.23), and patients with altered tendon structure or tendon pain exhibit higher total cholesterol, LDL–C, and triglycerides but lower HDL–C [27, 28]. In contrast, our acute postoperative setting captured ApoA-I levels before surgical trauma, and elevated baseline ApoA-I may instead reflect a distinct metabolic or inflammatory phenotype that predisposes to heightened pain sensitivity following surgical injury, rather than serving as a direct mediator of pain.

Given the exploratory nature of this finding and the observational design of our study, we cannot establish a causal relationship between ApoA-I and postoperative pain. It remains possible that ApoA-I serves as a surrogate marker for an underlying pain-prone metabolic or inflammatory state, rather than a direct mediator of pain. For example, patients with higher ApoA-I levels may have distinct systemic inflammatory profiles or metabolic phenotypes that predispose them to heightened pain sensitivity following surgical injury. Our observation should therefore be considered hypothesis-generating, and further prospective mechanistic studies are warranted to elucidate the underlying pathways and to determine whether preoperative ApoA-I measurement could be useful for stratifying patients for intensified pain management.

Our study did not find a statistically significant association between apolipoprotein C levels and postoperative pain after adjustment, although univariate analysis showed a difference (*P* = 0.046). Previous studies have found increased apolipoprotein C1 (APOC1) levels in cerebrospinal fluid of patients with neuropathic pain and a trend toward elevation in fibromyalgia, suggesting that APOC1 may be involved in central pain processing mechanisms [15]. The discrepancy between their findings and ours may reflect differences in biological compartment (CSF vs. peripheral blood) and pain type (chronic central sensitization vs. acute nociceptive postoperative pain). Further investigation of apolipoprotein C isoforms in acute surgical pain settings is warranted.

In summary, our findings suggest that the absence of a brachial plexus block and higher preoperative ApoA-I levels were associated with moderate-to-severe pain after shoulder arthroscopy. These results highlight the importance of individualized perioperative pain management strategies based on both surgical plans and patient-specific characteristics, including metabolic profiles. However, given the observational nature of this study, these findings should be confirmed by prospective randomized controlled trials with standardized analgesic protocols.

We acknowledge several important limitations that should be considered when interpreting our findings.

First, this is a retrospective, single-center observational study. Therefore, our analysis can only establish associations, not causality. The finding that “absence of nerve block” is a risk factor is particularly susceptible to confounding by indication. The decision to perform a nerve block was not randomized; patients who did not receive a block may have had contraindications, coexisting conditions, or more complex surgical requirements that could also have influenced their pain levels. Despite our efforts in statistical adjustment, the possibility of residual confounding from unmeasured or imprecisely measured confounders cannot be eliminated.

Second, the pain outcome measurement is limited. Although we extended pain assessments to include additional time points during hospitalization until discharge, the VAS score at 24 h after surgery remained the primary endpoint for analysis because it was the most consistently recorded time point across all patients. We assessed pain using this single VAS score dichotomized at VAS ≤3 versus >3, which does not fully capture the dynamic nature of postoperative pain, and the dichotomization may reduce statistical power. Additionally, VAS is inherently subjective, was assessed at rest rather than during movement, and was not evaluated by an observer blinded to nerve block status. The analgesic protocol was not fully standardized, and we did not collect detailed data on postoperative rescue analgesia, which may have influenced the reported pain scores. The heterogeneity of pain assessment methods is another factor that limits comparability across studies. A systematic review of 47 studies on arthroscopic rotator cuff surgery [27] found that although all studies measured pain intensity using a rating scale, the instruments varied considerably: 26 studies used the VAS, 17 used the NRS, 4 used a Likert scale, and 1 used a combined VAS and Faces Pain Scale. Recording time points and maximum-pain anchors were also inconsistent, with 28 different time points on postoperative day 1 and 14 different maximum-pain anchors identified. Only 11 of 47 studies specified whether pain was measured at rest or during activity, and analgesic consumption was reported using heterogeneous methods. This lack of standardization complicates systematic reviews and meta-analyses and may partly explain inconsistent conclusions across studies. In the present study, although using the VAS at 24 h as the primary endpoint ensured consistency across groups, the single-time-point dichotomization may, as noted in the limitations, fail to capture the dynamic nature of postoperative pain. Future studies may benefit from standardized assessment recorded both at rest and during activity.

Third, although patients were included consecutively, the study did not systematically exclude or account for other known critical confounders, such as preoperative pain intensity, chronic opioid use, psychiatric comorbidities, or failed/incomplete nerve blocks. These factors have been shown to influence postoperative pain in previous studies [4, 5] and may have contributed to the observed differences between the two pain groups. However, due to the retrospective design, these variables were not consistently documented in all patient records and could not be reliably extracted.

Fourth, regarding the apolipoprotein A-I (ApoA-I) measurement, blood samples were collected on the day before surgery after an overnight fast, which is a strength of our study as it eliminates concerns about reverse causation. However, we only measured ApoA-I at a single time point; we did not obtain postoperative measurements to examine whether ApoA-I levels change in response to surgical stress or pain, nor did we measure other related biomarkers (*e.g.*, inflammatory cytokines) that could help elucidate the underlying mechanism.

Fifth, our findings are derived from a specific population at a single institution in China, which may limit their generalizability to other centers, different patient demographics, or varying clinical practice patterns.

Finally, the sample size was relatively modest (*n* = 272) and was not based on a formal power calculation. While we identified several statistically significant associations, the limited sample size may have reduced our ability to detect smaller but clinically meaningful differences or to include more variables in the multivariable regression model without overfitting.

Despite these limitations, our findings contribute preliminary data on an under-studied association and support the need for a well-designed, prospective, multi-center study with a standardized analgesic protocol to confirm the role of both ApoA-I and regional anesthesia techniques in postoperative shoulder arthroscopy pain.

## Conclusion

This retrospective study of 272 patients undergoing shoulder arthroscopy found that the incidence of moderate-to-severe pain at 24 h after surgery was as high as 71.32%. The absence of an interscalene brachial plexus block and higher preoperative apolipoprotein A-I levels appeared to be associated with moderate-to-severe postoperative pain. These findings suggest that regional block techniques and preoperative metabolic characteristics, particularly ApoA-I levels, may deserve consideration in perioperative pain management for shoulder arthroscopy. However, given the observational design of this study, these associations should be interpreted with caution and need to be confirmed by prospective randomized controlled trials. A graphical summary of the study design, key findings, and clinical implications is presented in Figure 3.

**Figure 3:**
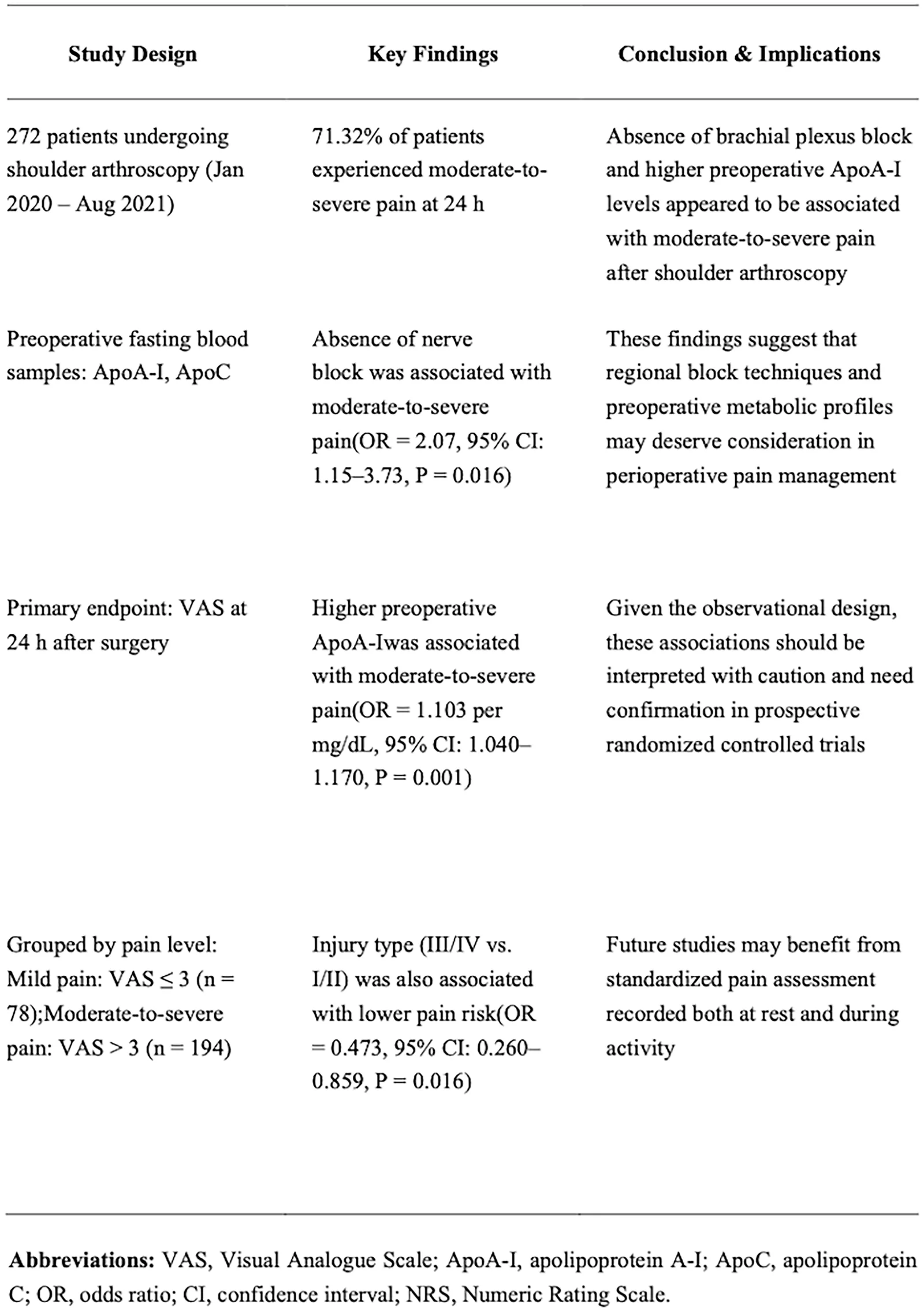
Graphical abstract. Summary of study design, key findings, and clinical implications regarding factors associated with moderate-to-severe pain after shoulder arthroscopy.

## Data Availability

De-identified data are available upon reasonable request from the corresponding author, pending institutional review board approval.
